# Predicting drug side effects by multi-label learning and ensemble learning

**DOI:** 10.1186/s12859-015-0774-y

**Published:** 2015-11-04

**Authors:** Wen Zhang, Feng Liu, Longqiang Luo, Jingxia Zhang

**Affiliations:** 1 School of Computer, Wuhan University, Wuhan, 430072 China; 2 Research Institute of Shenzhen, Wuhan University, Shenzhen, 518057 China; 3 International School of software, Wuhan University, Wuhan, 430072 China; 4 School of Mathematics and Statistics, Wuhan University, Wuhan, 430072 China

**Keywords:** Side effects, Multi-label learning, Ensemble learning

## Abstract

**Background:**

Predicting drug side effects is an important topic in the drug discovery. Although several machine learning methods have been proposed to predict side effects, there is still space for improvements. Firstly, the side effect prediction is a multi-label learning task, and we can adopt the multi-label learning techniques for it. Secondly, drug-related features are associated with side effects, and feature dimensions have specific biological meanings. Recognizing critical dimensions and reducing irrelevant dimensions may help to reveal the causes of side effects.

**Methods:**

In this paper, we propose a novel method ‘feature selection-based multi-label k-nearest neighbor method’ (FS-MLKNN), which can simultaneously determine critical feature dimensions and construct high-accuracy multi-label prediction models.

**Results:**

Computational experiments demonstrate that FS-MLKNN leads to good performances as well as explainable results. To achieve better performances, we further develop the ensemble learning model by integrating individual feature-based FS-MLKNN models. When compared with other state-of-the-art methods, the ensemble method produces better performances on benchmark datasets.

**Conclusions:**

In conclusion, FS-MLKNN and the ensemble method are promising tools for the side effect prediction. The source code and datasets are available in the Additional file 1.

**Electronic supplementary material:**

The online version of this article (doi:10.1186/s12859-015-0774-y) contains supplementary material, which is available to authorized users.

## Background

Drugs can help to treat diseases, but usually come with side effects or adverse reactions. Because of unintended side effects, a great number of approved drugs were even withdrawn from the market. Therefore, recognizing potential side effects helps to reduce costs and avoid risks in the drug discovery. However, wet experiments are costly and time-consuming. Since researchers collected drug data and compile them in the public databases, computational methods were developed for the side effect prediction.

The traditional computational methods analyzed the structure-activity relationship or quantitative structure–property relationship [1–5], but they are not suitable for the large-scale data. In recent years, machine learning methods were applied to the drug side effect prediction, because of their capability of dealing with complicated data. Huang [6] combined drug targets, protein-protein interaction networks and gene ontology annotations, and then respectively adopted the support vector machine (SVM) and logistic regression to build prediction models. Pauwels [7] considered chemical substructures of drug candidate molecules, and respectively adopted four machine learning methods (k-nearest neighbor, support vector machine, ordinary canonical correlation analysis and sparse canonical correlation analysis) to construct prediction models. Yamanishi [8, 9] combined the chemical substructures and target protein information about drugs, and adopted the sparse canonical correlation analysis for prediction. Liu [10] integrated the phenotypic information, chemical information and biological information about drugs, and then built the prediction models by using different machine learning classifiers (logistic regression, naive Bayes, k-nearest neighbor, random forest and SVM). Bresso [11] adopted the decision trees and inductive logic programming to identify and characterize side-effect profiles shared by several drugs. Cheng [12] proposed a phenotypic network inference-based method. Huang [13] integrated protein-protein interaction networks and drug substructures, and built SVM-based models. Liu [14] determined molecular predictors of adverse drug reactions with causality analysis.

Although several machine learning methods have been proposed to predict side effects, there is still space for improvements. Firstly, the side effect prediction is actually a multi-label learning task, but far less attention has been paid to this point. Therefore, we make efforts to solve the problem in the frame of multi-label learning. Although lots of multi-label learning methods have been proposed, they can’t be directly used for our task, which have thousands of labels and severely imbalanced data. Secondly, several drug-related features are associated with side effects, and dimensions of each feature are biological components. For example, there are 881 types of substructures described by PubChem. Since a drug may have specific substructures, it is represented by an 881-dimensional feature vector, in which ‘0’ or ‘1’ means the absence or presence of the corresponding substructure. However, not all substructures are necessarily related with side effects, and some may be redundant. Therefore, identifying critical feature dimensions or reducing irrelevant dimensions can help to investigate the cause of side effects, and thus probably improve predictive performances.

In this paper, we propose a novel method named ‘feature selection-based multi-label k-nearest neighbor method’ (FS-MLKNN) for the side effect prediction. FS-MLKNN takes two steps to build the relationship between feature vectors and side effects. In the first step, informative dimensions are selected by using mutual information between feature dimensions and side effects, so as to reduce the computational burden of multi-label learning. In the second step, the genetic algorithm (GA) and the multi-label k-nearest neighbor method (MLKNN) are further combined to determine the optimal feature dimensions and develop the prediction model. Computational experiments demonstrate that FS-MLKNN can produce high-accuracy performances.

To the best of our knowledge, various features may bring diverse information as well as noise. The problem is how to effectively integrate them. Here, we adopt ensemble learning techniques to combine various features. Specifically, individual feature-based models are constructed by FS-MLKNN and used as base predictors, and the ensemble strategy named ‘weighted scoring’ is used to integrate base predictors and develop the ensemble models. The computational results show that the ensemble method can make improvements, and lead to better performances than other state-of-the-art methods. The source code and datasets are available in the Additional file 1.

## Methods

### Datasets

There are several public databases about drugs, side effects and related information. SIDER database [15] contains information on marketed medicines and their adverse drug reactions. PubChem Compound database [16, 17] contains validated chemical information. DrugBank database [18–21] is a bioinformatics resource that combines detailed drug data with comprehensive drug target information. KEGG DRUG [22] is a drug information resource for approved drugs in Japan, USA and Europe.

A variety of drug-related features can be obtained from these databases and are considered to be associated with side effects. The drug chemical substructures are usually considered as the most important factor for drug side effects. Drug targets are usually involved in a particular metabolic or signaling pathway, and may provide the important clue to drug side effects. Drug transporters are expressed in many tissues, and play key roles in drug absorption, distribution, and excretion. Drugs usually undergo drug metabolism to be biologically active, and the enzymes may influence the metabolism thus induce side effects. The unintended biochemical pathways and drug indications may cause most drug side effects.

To the best of our knowledge, several datasets were ever used in the previous studies and now publicly available, i.e. Pauwels’s dataset [7], Mizutani’s dataset [8] and Liu’s dataset [10]. Hence, we use them as the benchmark datasets, and the details of datasets are described in Table 1. Since Liu’s dataset contains a variety of features (substructures, enzymes, pathways, targets, transporters and indications), we use it to discuss the usefulness of various features and demonstrate the advantages of FS-MLKNN. Previous state-of-the-art methods were constructed on the Pauwels’s dataset [7], Mizutani’s dataset [8] and Liu’s dataset [10], and we construct our models on the same datasets to make comparison.

**Table 1:** The details about benchmark datasets

Dataset	#drug	#side effect	#substructure	#target	#transporter	#enzyme	#pathway	# indication
Pauwels’s dataset	888	1385	881	N.A	N.A	N.A	N.A	N.A
Mizutani’s dataset	658	1339	881	1368	N.A	N.A	N.A	N.A
Liu’s dataset	832	1385	881	786	72	111	173	869

*N.A*. means these features are not included in the datasets

Recently, SIDER database has been updated for the fourth edition, which contains 1430 drugs and 5880 side effect terms. According to PubChem ID, CAS registry number, IUPAC International Chemical Identifier (InChI key) or drug name, 1080 SIDER drugs are successfully mapped to DrugBank database, which contains a variety of drug-related biological features. Moreover, we remove side effect terms which are associated with more than 3 drugs. Finally, we compile a dataset with 1080 drugs and 2260 side effect terms, which we name ‘SIDER 4 dataset’. The features (substructures, enzymes, pathways, targets, transporters and indications) for these drugs are included in the dataset. In SIDER 4 dataset, 771 drugs are included in the initial edition of SIDER database and Liu’s dataset, and the others (309) are newly added drugs. SIDER 4 dataset is used for the independent experiment.

### The multi-label learning for side effect prediction

#### Problem formulation

In the side effect prediction, prediction models are constructed on the training drugs, and are applied to the testing drugs. Formally, multi-label learning is to build a model that maps inputs to binary vectors, rather than scalar outputs of the ordinary classification. Since a drug is usually associated with multiple side effects, the work can be formulated as a multi-label classification problem.

Representation of drug features and drug labels is the first step in the multi-label learning. There are a variety of features for drugs, such as chemical substructures, target proteins, indications, etc. Each feature can help to represent a drug as a feature vector, and the dimensions are binary values 1 or 0, which mean the presence or absence of corresponding components. As mentioned above, the ‘substructure’ (described in PubChem) feature vector of a drug is 881-dimensional. Similarly, side effects of a drug are represented by a binary vector, in which values ‘1’ or ‘0’ means whether or not the drug induces corresponding side effects.

**Fig. 1:**
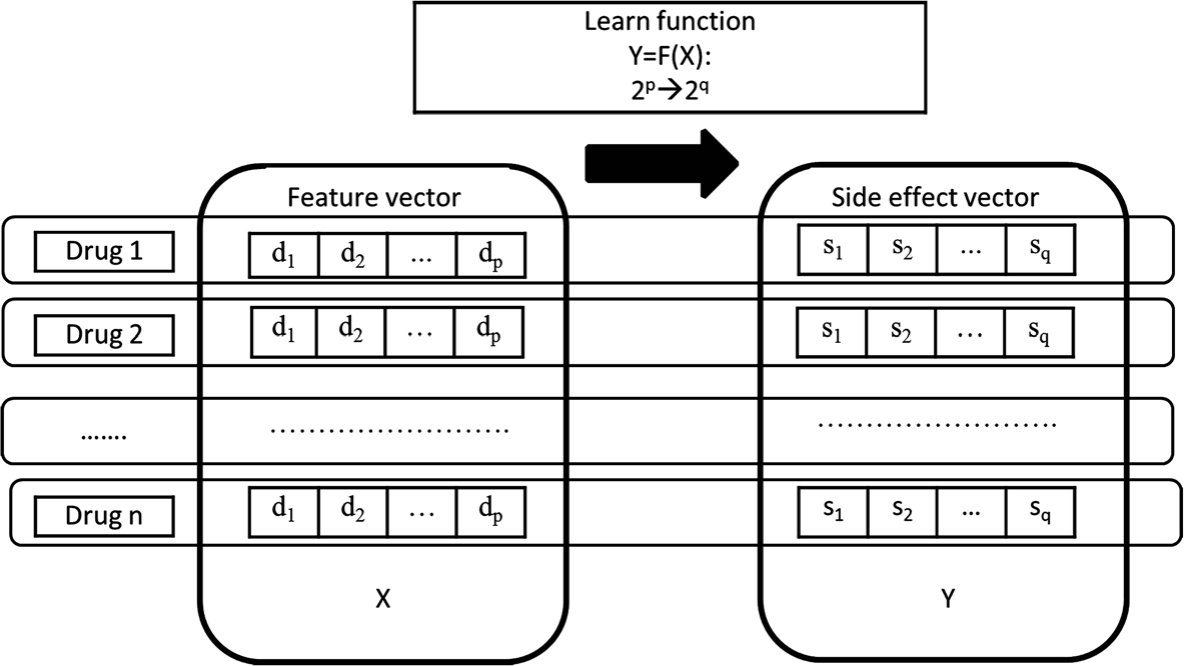
Multi-label task for drug side effect prediction and the data representation

Given a dataset of *n* drugs denoted as {(*x*_*i*_, *y*_*i*_)}_*i* = 1_^*n*^, *x*_*i*_ and *y*_*i*_ are the *p*-dimensional feature vector and *q*-dimensional side effect vector for the *i*th drug. As shown in Fig. 1, our goal is to build the functional relationship *Y* = *F*(*X*) : 2^*p*^ → 2^*q*^ between exploratory variables (feature vector) and target values (side effect vector) for multi-label learning.

Since this work makes predictions for thousands of side effects (classification labels), we should take into account the accuracy as well as efficiency. Here, we propose the method named ‘feature selection-based multi-label k-nearest neighbor method’ (FS-MLKNN). The classic method ‘multi-label k-nearest neighbor’ (MLKNN) [23] is the core of FS-MLKNN. By inheriting advantages of MLKNN, FS-MLKNN can produce high-accuracy performances for our task, and more importantly give out the explainable results. The reasons for adopting MLKNN are as follows. The multi-label learning algorithms are roughly divided into two types: transformation methods and adaption methods [24, 25]. Transformation methods usually transform the multi-label problem into a set of binary classification problems; adaption methods directly perform the multi-label classification. The study in [25] compared a great number of multi-label learning methods, and the experiments demonstrated that the performances of MLKNN are close to that of the best methods (RF-PCT and HOMER). The study in [26, 27] showed that most existing multi-label learning algorithms will fail when the label space is large, e.g. number of labels > 50. In contrast, MLKNN can deal with thousands of side effect labels in a reasonable amount of time.

### Multi-label k-nearest neighbor method

Based on notations in Fig. 1, we briefly introduce MLKNN. Given the training set {*x*_*i*_, *y*_*i*_}_*i* = 1_^*n*^, *x*_*i*_ is the *i*th instance (drug), and *y*_*i*_ is the corresponding side effect vector. *y*_*i*_(*l*) = 1, if the *i*th instance can induce the *l* th side effect, otherwise and *y*_*i*_(*l*) = 0, *l* = 1, 2, ⋯, *q*. The *k* nearest neighbors (in training set) of instance *x*_*i*_ are denoted by *N*(*x*_*i*_), *i* = 1, 2, ⋯, *n*. Thus, based on the *l*th side effect of these neighbors, a membership counting vector can be denoted as:

$$ {C}_{x_i}(l)={\displaystyle {\sum}_{a\in N\left({x}_i\right)}{y}_a(l),\kern0.4em l=1,2,\cdots, q} $$

where ${C}_{x_i}(l)$ counts the number of neighbors of *x*_*i*_ inducing the *l*th side effect, and $0\le {C}_{x_i}(l)\le k$.

For a test drug *t*, MLKNN identifies its *k* nearest neighbors in the training set, and calculate *C*_*t*_(*l*). Let *H*_1_^*l*^ be the event that a drug has *l*th side effect and *H*_0_^*l*^ be the event that a drug does not has *l*th side effect. Let *E*_*j*_^*l*^ be the event that a drug just has *j* neighbors with *l*th side effect in its *k* nearest neighbors. For the instance *t*, its label for *l*th side effect *y*_*t*_(*l*) is determined by the following principle:

$$ {y}_t(l)= \arg \kern0.3em { \max}_{b\in \left\{0,1\right\}}P\left({H}_b^l\Big|{E}_{C_t(l)}^l\right),\kern0.7em l=1,2,\cdots, q $$

Using the Bayesian rule, above Eq. can be rewritten as:

$$ {y}_t(l)= \arg \kern0.3em { \max}_{b\in \left\{0,1\right\}}\frac{P\left({H}_b^l\right)P\left({E}_{C_t(l)}^l\Big|{H}_b^l\right)}{P\left({E}_{C_t(l)}^l\right)}= \arg \kern0.3em { \max}_{b\in \left\{0,1\right\}}P\left({H}_b^l\right)P\left({E}_{C_t(l)}^l\Big|{H}_b^l\right) $$

In the prediction model, *P*(*H*_*b*_^*l*^) and *P*(*E*_*j*_^*l*^|*H*_*b*_^*l*^) are calculated based on the training set. The prior probabilities are calculated,

$$ P\left({H}_1^l\right)=\left(s,+,{\displaystyle {\sum}_{i=1}^n{y}_i(l)}\right)/\left(s\times 2+n\right)\operatorname{and}\kern0.4em P\left({H}_0^l\right)=1-P\left({H}_1^l\right) $$

Then, the posterior probabilities $P\left({E}_{C_{x_i}(l)}^l\Big|\kern0.2em {H}_0^l\right)$, $P\left({E}_{C_{x_i}(l)}^l\Big|\kern0.2em {H}_1^l\right)$ are calculated by following equations,

$$ P\left({E}_j^l\Big|\kern0.2em {H}_1^l\right)=\left(s+c\left[j\right]\right)/\left(s\times \left(k+1\right)+{\displaystyle {\sum}_{i=0}^k{c}_l\left[i\right]}\right) $$

$$ P\left({E}_j^l\left|{H}_0^l\right.\right)=\left(s+{c}^{\hbox{'}}\left[j\right]\right)/\left(s\times \left(k+1\right)+{\displaystyle {\sum}_{i=0}^k{c}_l^{\hbox{'}}\left[i\right]}\right) $$

$$ l=1,2,\cdot \cdot \cdot, q,\kern0.4em j=1,2,\cdot \cdot \cdot, k $$

Where *s* is the smooth factor. c_*l*_[*i*] is the number of instances which just have *i* neighbors with *l*th side effect in their *k* nearest neighbors; *c*_*l*_^'^[*i*] is the number of instances which just have *i* neighbors without *l*th side effect in their *k* nearest neighbors.

### Feature selection-based multi-label k-nearest neighbor method

**Fig. 2:**
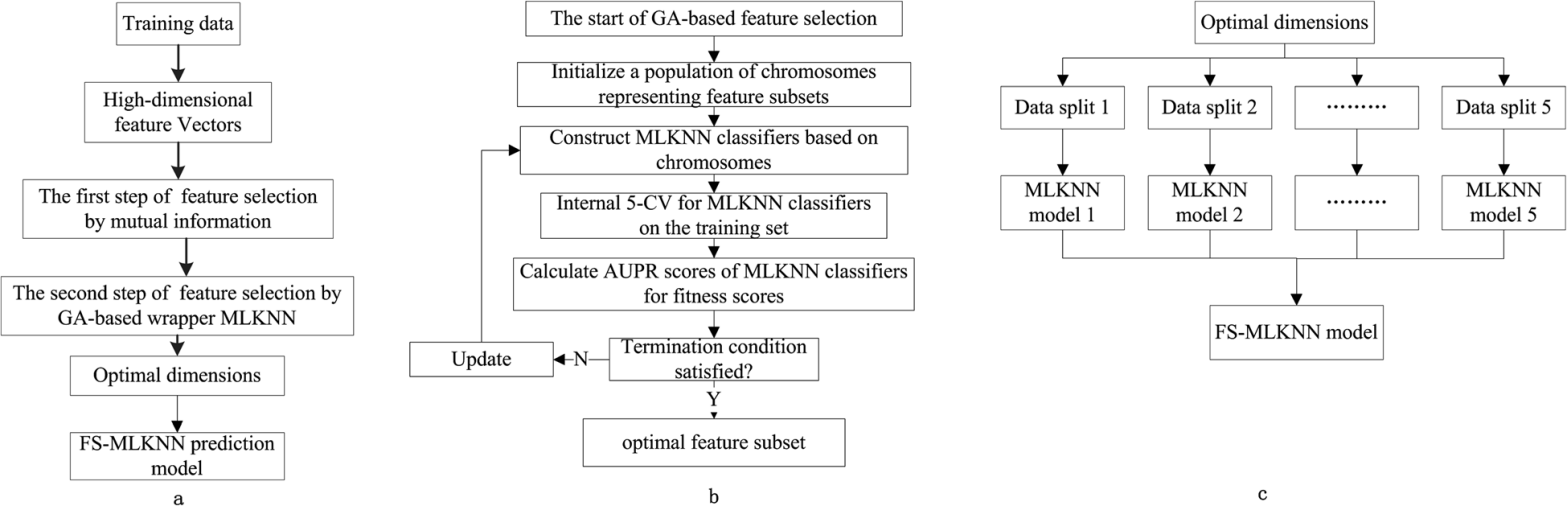
**a** Flowchart of FS-MLKNN **b** details about the GA-based wrapper feature selection **c** the details about constructing FS-MLKNN prediction model

We design the feature selection-based multi-label k-nearest neighbor method (FS-MLKNN) to simultaneously determine the optimal feature dimensions and build multi-label prediction models. Here, *p* dimensions of feature vectors and *q* dimensions of side effect vectors are respectively denoted as *V* = {*v*_1_, *v*_2_, ⋅ ⋅⋅, *v*_*p*_} and *D* = {*d*_1_, *d*_2_, ⋅ ⋅⋅, *d*_*q*_}. As shown in Fig. 2(a), FS-MLKNN has two steps.

In the first step, we use mutual information to select critical dimensions of the feature. The mutual information between the feature dimension *v*_*i*_ and the label *d*_*j*_ is calculate as,

$$ I\left({v}_i;{D}_j\right)={\displaystyle \sum_{x\in {v}_i}{\displaystyle \sum_{y\in {d}_j}p\left(x,y\right) \log \frac{p\left(x,y\right)}{p(x)p(y)}},i=1,2,\cdots, p,\kern0.5em j=1,2,\cdots, q} $$

We define the correlation between the feature dimension *v*_*i*_ and the label set *D* = {*d*_1_, *d*_2_, ⋯, *d*_*q*_},

$$ I\left({v}_i;D\right)=I\left({v}_i;{d}_1;,;{d}_2;,;\cdots;,; {d}_q\right)=\underset{i\in \left\{1,2,..q\right\}}{ \max }I\left({v}_i\Big|{d}_j\right) $$

Then, the dimensions subset *V* ^'^ = {*v*_*i*_ | *I*(*v*_*i*_; *D*) > *σ*} is selected, and then used in the next step.

In the second step, we combine MLKNN and genetic algorithm (GA) to select the optimal dimension subset and develop multi-label classification model. GA is a search approach that mimics the process of natural selection. GA can effectively search the interesting space and easily solve complex problems without requiring the prior knowledge about the space and the problem. As shown in Fig. 2(b), the dimension subsets are encoded as chromosomes and then form a population. MLKNN is adopted as the multi-learning engine, and MLKNN models are respectively constructed based on the dimension subsets. Then, models are evaluated by the internal 5-fold cross validation on the training data, and AUPR scores are used as the fitness scores of corresponding chromosomes. The population is updated by selection, mutation and variance. The optimal dimension subset is finally determined by internal 5-CV on the training set, and then we respectively build five MLKNN models based on the training data in five internal folds and selected dimensions. As shown in Fig. 2(c), the FS-MLKNN model consists of five MLKNN models. When making predictions for the testing set, the average scores of outputs by five MLKNN models are adopted as the predictions of FS-MLKNN model.

### Combining various features by ensemble learning

There are different drug-related features for side effect prediction, and each feature can be used to encode drugs and thus develop FS-MLKNN models. Here, we attempt to combine various features to achieve better performances.

In machine learning, the work that combines various features is also known as feature fusion, whose purpose is to exploit features and remove the redundant information. In bioinformatics, merging various feature vectors is a simple and widely used feature fusion approach, but it may be affected by the redundant information between features. Ensemble learning is a sophisticated technique of combining features, which recently attracts more and more interests in bioinformatics. The success of ensemble learning has been proved by lots of applications [28–33].

**Fig. 3:**
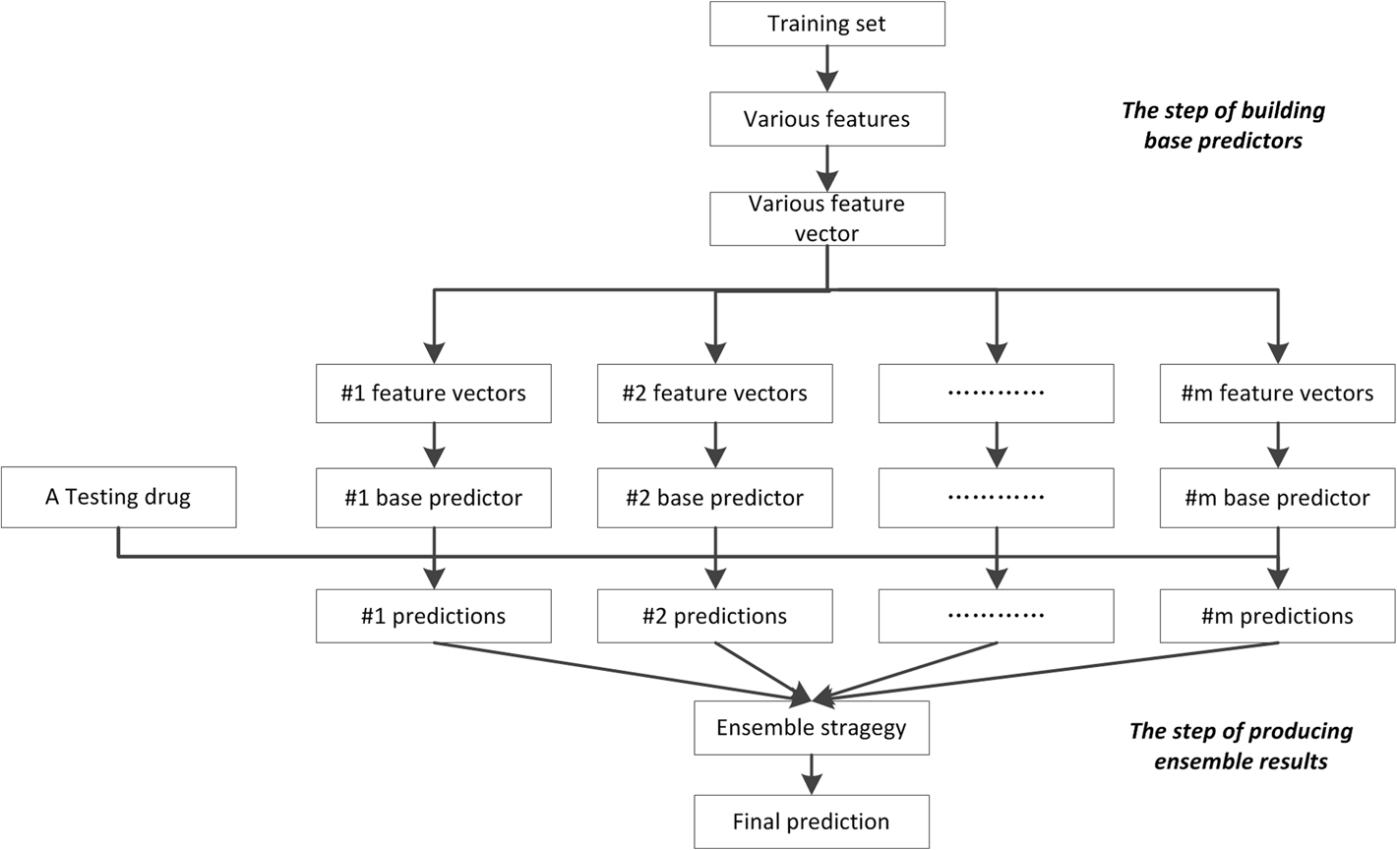
Flowchart of ensemble learning

Here, we design the ensemble learning method to combine various features and develop high-accuracy prediction models. Figure 3 shows the flowchart of the ensemble method. Given *m* features, we build *m* individual feature-based FS-MLKNN models, and use them as base predictors. Since features may make different contributes, it is natural to adopt weighted scoring ensemble strategy, which assigns *m* base predictors with *m* weights {*w*_1_, *w*_2_, ⋯, *w*_*m*_}. For a testing instance, the *i*th predictor will give scores for *q* side effects, denoted as *S*_*i*_ = {*s*_*i*_^1^, *s*_*i*_^2^, ⋯, *s*_*i*_^*q*^}, *i* = 1, 2, ⋯, *m*. The final prediction produced by the ensemble model is the linear weighted sum of outputs from base predictors,

$$ Ensemble\kern0.5em  Score=\left[{w}_1,{w}_2,\cdot \cdot \cdot, {w}_m\right]\times \left[\begin{array}{c}\hfill {S}_1\hfill \\ {}\hfill {S}_2\hfill \\ {}\hfill \cdot \cdot \cdot \hfill \\ {}\hfill {S}_m\hfill \end{array}\right]=\left[{w}_1,{w}_2,\cdot \cdot \cdot, {w}_m\right]\times \left[\begin{array}{cccc}\hfill {S}_1^1\hfill & \hfill {S}_1^2\hfill & \hfill \cdot \cdot \cdot \hfill & \hfill {S}_1^q\hfill \\ {}\hfill {S}_2^1\hfill & \hfill {S}_2^2\hfill & \hfill \cdot \cdot \cdot \hfill & \hfill {S}_2^q\hfill \\ {}\hfill \cdot \cdot \cdot \hfill & \hfill \cdot \cdot \cdot \hfill & \hfill \cdot \cdot \cdot \hfill & \hfill \cdot \cdot \cdot \hfill \\ {}\hfill {S}_m^1\hfill & \hfill {S}_m^2\hfill & \hfill \cdot \cdot \cdot \hfill & \hfill {S}_m^q\hfill \end{array}\right] $$

Tuning weights for base predictors are critical for the ensemble models. The weights are non-negative real values between 0 and 1, and the sum of weights equals 1. We adopt the genetic algorithm to search for the optimal weights, and the internal 5-CV AUPR on training data is used as the fitness score.

### Experimental configurations and evaluation metrics

Here, 5-fold cross validation (5-CV) is adopted to test performances of models. For a dataset, all drugs are randomly split into five subsets with equal size. Each time, four subsets are combined as the training set, and the remaining subset is used as the testing set. The models are trained on the training drugs and their side effects, and then are used to predict side effects of testing drugs. The procedure is repeated, until each subset has been ever used for testing.

In the side effect prediction, the predicted scores for side effects were usually merged for evaluation, and the metrics for ordinary binary classification were often adopted. The area under ROC curve (AUC) and the area under the precision-recall curve (AUPR) can be used to evaluate models regardless of any threshold. However, there are much more negative labels than positive labels in the side effect prediction, and machine-learning methods are likely to produce overestimated AUC scores. Since AUPR takes into account recall as well as precision, it is used as the most important metric. To the best of our knowledge, several metrics were designed for multi-learning classification [24], i.e. Hamming loss, one-error, coverage, ranking loss and average precision. Hamming loss is the fraction of the wrong labels to the total number of labels. The one-error evaluates the fraction of examples whose top-ranked label is not in the relevant label set. The coverage evaluates how many steps are needed, on average, to move down the ranked label list so as to cover all the relevant labels of the example. The average precision evaluates the average fraction of relevant labels ranked higher than a particular label. Therefore, we adopt AUPR, average precision, one-error, coverage, ranking loss and hamming loss for the side effect prediction.

For one-error, coverage, ranking loss and hamming loss, the smaller the metric value the better the system’s performance. For AUPR and average precision, the larger the metric value the better the system’s performance.

We have to set parameters for FS-MLKNN. In the first step of FS-MLKNN, the threshold 0.001 is used for the mutual information-based feature selection. In the second step, Matlab genetic algorithm toolbox is used to implement GA optimization, and the default parameters are adopted for genetic operator, crossover operator and mutation operator. The population of GA is set as 100. The population update will terminate when the change of best fitness scores is less than the default value of 1E-6 or the max generation number of 60 is reached. The same configurations are used for the GA optimization in weighted scoring ensemble learning.

## Results and discussion

### Performances of FS-MLKNN

First of all, the experiments are carried out on Liu’s dataset to investigate the usefulness of various features and demonstrate the advantages of FS-MLKNN. Specifically, we encode the drugs with individual features, and then respectively adopt FS-MLKNN and MLKNN as the multi-label learning engines to construct prediction models. To provide a comprehensive evaluation of the method, we randomly repeat the data separation, and implement 5 runs of 5-cross validation for FS-MLKNN models and MLKNN models. The means and standard deviations of metric scores are respectively demonstrated in Table 2 and Table 3.

**Table 2:** The 5-CV performances of individual feature-based MLKNN models on Liu’s dataset

Features	AUC	AUPR	Hamming loss	Ranking loss	One error	Coverage	Average precision
Enzyme	0.8861 ± 0.0006	0.3989 ± 0.0011	0.0483 ± 0.0001	0.0839 ± 0.0002	0.1695 ± 0.0053	837.7197 ± 1.6124	0.4551 ± 0.0005
Pathway	0.8884 ± 0.0006	0.4105 ± 0.0010	0.0477 ± 0.0001	0.0802 ± 0.0001	0.1865 ± 0.0076	827.1183 ± 2.9986	0.4721 ± 0.0007
Target	0.8947 ± 0.0009	0.4424 ± 0.0017	0.0464 ± 0.0001	0.0745 ± 0.0003	0.1695 ± 0.0061	812.6752 ± 2.9022	0.4919 ± 0.0010
Transporter	0.8863 ± 0.0006	0.4010 ± 0.0013	0.0482 ± 0.0001	0.0826 ± 0.0002	0.1661 ± 0.0041	836.2058 ± 2.8593	0.4644 ± 0.0007
Indication	0.8948 ± 0.0004	0.4566 ± 0.0020	0.0456 ± 0.0001	0.0762 ± 0.0003	0.1363 ± 0.0034	818.3745 ± 3.6611	0.4950 ± 0.0012
Substructure	0.8912 ± 0.0005	0.4255 ± 0.0015	0.0472 ± 0.0001	0.0754 ± 0.0004	0.1760 ± 0.0040	808.9192 ± 2.4440	0.4888 ± 0.0014

**Table 3:** The 5-CV performances of individual feature-based FS-MLKNN models on Liu’s dataset

Features	AUC	AUPR	Hamming loss	Ranking loss	One error	Coverage	Average precision
Enzyme	0.8878 ± 0.0004	0.4080 ± 0.0013	0.0478 ± 0.0001	0.0826 ± 0.0002	0.1611 ± 0.0057	837.1250 ± 2.9063	0.4652 ± 0.0005
Pathway	0.8895 ± 0.0006	0.4187 ± 0.0028	0.0473 ± 0.0001	0.0792 ± 0.0003	0.1688 ± 0.0037	824.2678 ± 4.2341	0.4799 ± 0.0006
Target	0.8962 ± 0.0007	0.4557 ± 0.0019	0.0457 ± 0.0001	0.0739 ± 0.0003	0.1442 ± 0.0048	810.4788 ± 2.9801	0.5008 ± 0.0008
Transporter	0.8871 ± 0.0008	0.4060 ± 0.0018	0.0480 ± 0.0001	0.0819 ± 0.0003	0.1635 ± 0.0037	836.4404 ± 2.3029	0.4698 ± 0.0007
Indication	0.8963 ± 0.0008	0.4648 ± 0.0043	0.0452 ± 0.0002	0.0755 ± 0.0003	0.1341 ± 0.0054	818.0483 ± 3.9917	0.5005 ± 0.0014
Substructure	0.8931 ± 0.0005	0.4343 ± 0.0011	0.0468 ± 0.0001	0.0739 ± 0.0005	0.1659 ± 0.0069	804.3813 ± 2.7354	0.4989 ± 0.0021

According to the standard deviations of metric scores, FS-MLKNN is likely to produce similar results for different splits of cross validation, and so does MLKNN. Therefore, we compare mean metric scores of FS-MLKNN and MLKNN based on the same features. The results in Table 2 and Table 3 show that FS-MLKNN produces better performances than MLKNN, no matter which features are used, and we can clearly observe the improvements on the AUPR scores. It is observed that the models produce overestimated AUC scores for the imbalanced data in the experiment, and AUC is not a suitable metric for the problem. Since MLKNN and FS-MLKNN are robust to the data split of the cross validation, we make analysis and comparison based on the same data split in the following content.

Further, we evaluate their predictions for individual side effect terms. For each side effect term, the predicted scores and real labels are used to calculate the AUPR score. Although there are 1385 side effect terms in Liu’s dataset, 4 side effect terms are not observed for any of 832 drugs in the dataset. Since there is no positive instance for these side effect terms, their AUPR scores can’t be calculated. Therefore, we can respectively obtain 1381 AUPR scores for 1381 side effect terms based on the results of FS-MLKNN and MLKNN. Then, we compare their AUPR scores for each side effect term. As shown in Table 4, we count the numbers of three cases (FS-MLKNN better than MLKNN, FS-MLKNN equals to MLKNN, MLKNN better than FS-MLKNN). For thousands of side effect terms, cases that FS-MLKNN is better than MLKNN are much more than the cases that MLKNN is better than FS-MLKNN. For six features, FS-MLKNN can consistently produce better performances than MLKNN.

**Table 4:** The comparison of side effect-based AUPR scores produced by FS-MLKNN and MLKNN

Features	# FS-MLKNN > MLKNN	# FS-MLKNN = MLKNN	# FS-MLKNN < MLKNN
Enzyme	501	553	327
Pathway	432	543	406
Target	492	537	352
Transporter	457	568	356
Indication	497	566	318
Substructure	447	508	426

**Fig. 4:**
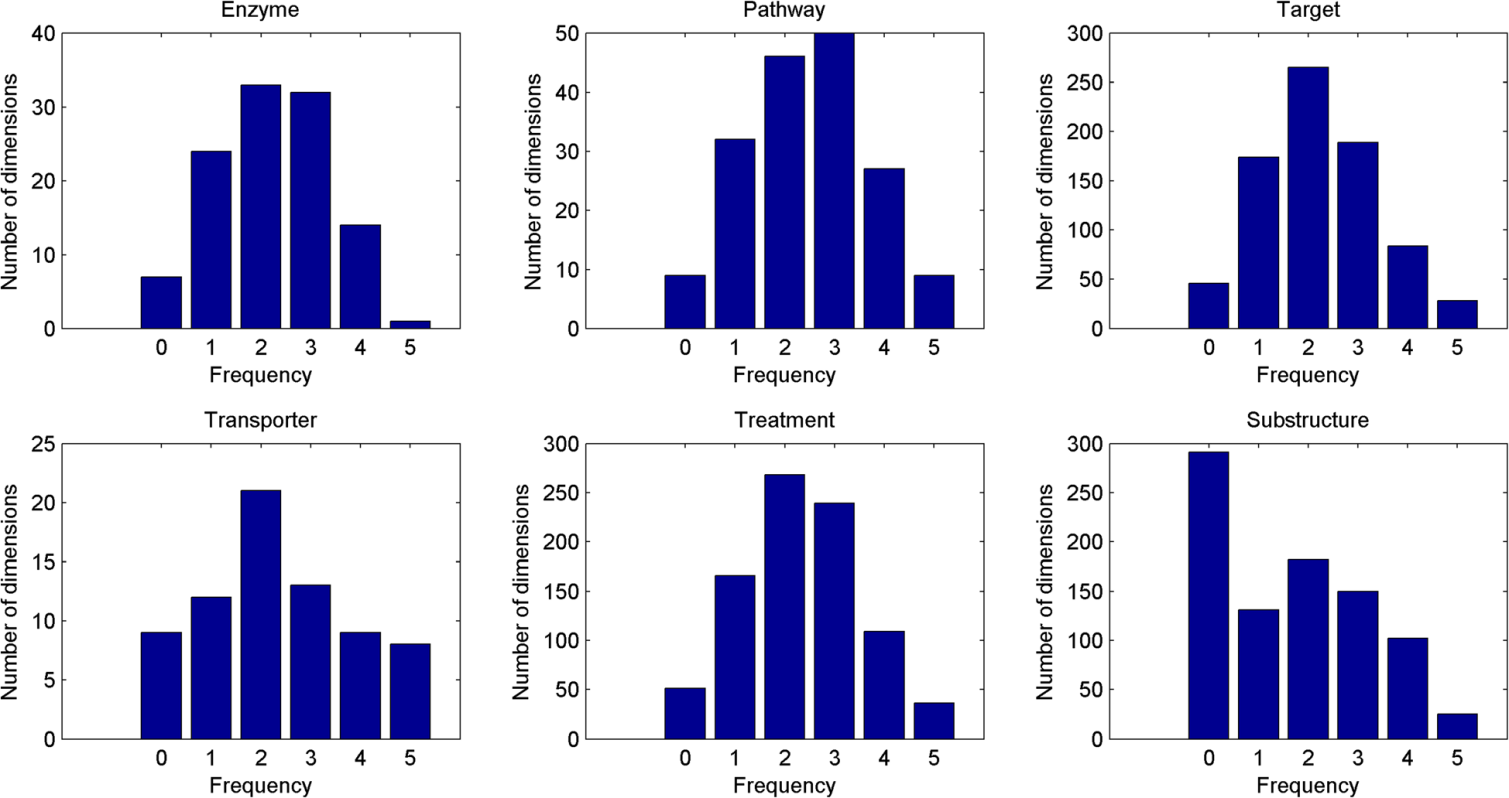
The frequencies of selected dimensions for various features (on Liu’s dataset). 0,1,2,3,4 mean the number of selected features in five folds; 5 means the number of features selected by all five folds

Although there are hundreds of dimensions for each feature, FS-MLKNN selects a small proportion of dimensions for prediction. The superior performances of FS-MLKNN are due to the removal of redundant feature dimensions. The optimal feature dimensions which are determined by FS-MLKNN are shown in Table 5. Since we implement 5-fold cross validation, there are five different sets of optimal dimensions for each model. We calculate the frequencies of selected dimensions in five folds, and the statistics are shown in Fig. 4. For feature ‘substructure’, a great proportion of the dimensions are never selected. In contrast, only a few dimensions are selected in all folds, for four features ‘enzyme’, ‘pathway’, ‘target’, ‘transporter’ and ‘indication’. The selected feature dimensions are analyzed in the following section ‘independent experiment and case study’.

**Table 5:** The dimensions determined by FS-MLKNN on Liu’s dataset

Feature	Original #	1-fold #	2-fold #	3-fold #	4-fold #	5-fold #	Average #
Enzyme	111	39	42	54	57	55	49
Pathway	173	88	74	96	88	81	85
Target	786	323	353	378	360	333	349
Transporter	72	33	34	33	41	28	34
Indication	869	407	413	414	392	409	407
Substructure	881	310	295	287	279	307	296

### Performances of ensemble models

The performances of individual feature-based methods can measure the usefulness of corresponding features. According to Table 2, these features can yield the AUPR scores around or greater than 0.4, and ‘indication’ can lead to the best results. The most possible reason is that the intents for which a drug is designed are usually associated with the induced side effects. In general, all features are of potential use for the side effect prediction.

Since different features provide important information in different views, we have to consider how to combine various features effectively. In this paper, we consider the weighted scoring ensemble learning to develop the final prediction models. Because of six drug-related features, six individual feature-based models are constructed by FS-MLKNN and used as base predictors. Weights are assigned to these base predictors, and linear weighted sums of outputs are adopted as the prediction by the ensemble method.

The genetic algorithm is used to tune weights in the ensemble method, and optimal weights are shown in Table 6. The weights reflect the contributions of features. The results show that the major contributions of predictions come from indication, target and substructure, and the contributions of enzyme, pathway, and transport are much smaller. In general, weights have relation with performances of individual feature-based models (Table 3), and the features which produce better results are given greater weights in the ensemble model.

**Table 6:** The weights of ensemble models on Liu’s dataset

	Enzyme	Pathway	Target	Transporter	Indication	Substructure
1-fold	0.009	0.002	0.265	0.023	0.515	0.185
2-fold	0.012	0.104	0.210	0.009	0.489	0.174
3-fold	0.060	0.042	0.370	0.000	0.458	0.069
4-fold	0.001	0.035	0.261	0.037	0.481	0.185
5-fold	0.001	0.035	0.261	0.037	0.481	0.185
Average	0.017	0.044	0.273	0.021	0.485	0.160

**Fig. 5:**
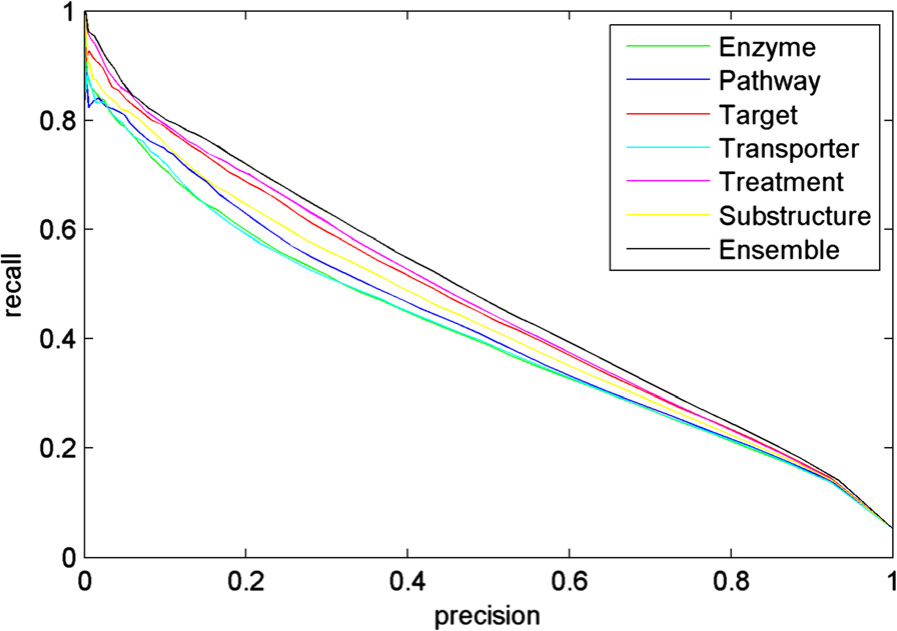
AUPR curves of individual feature-based models and the ensemble model on Liu’s dataset. Enzyme (0.4078), pathway (0.4210), target (0.4575), transporter (0.4085), indication (0.4646), substructure (0.4362) and ensemble method (0.4802)

Further, we compare the performances of the ensemble models with that of individual feature-based FS-MLKNN models. As shown in Fig. 5, the ensemble model can make improvements over individual feature-based models in terms of AUPR. Compared with the best model based on the feature ‘indication’ (AUPR score of 0.4646 on Liu’s dataset), the ensemble method yields the higher AUPR score of 0.4802. There are some possible reasons for the improvements. Firstly, a variety of features provide diverse information for ensemble learning. Secondly, different weights are assigned to features, so as to consider their different contributions.

### Comparison with benchmark methods

To the best of our knowledge, some state-of-the-art methods, i.e. Pauwels’s method [7], Mizutani’s method [8], Liu’s method [10] and Cheng’s method [12], were proposed for side effect prediction, and their source codes or datasets are publicly available.

These methods are roughly of two sorts: the side effect profile prediction methods and the potential side effect prediction methods. The side effect profile prediction methods (Pauwels’s method and Mizutani’s method) attempt to predict all interested side effects for a drug, and predicted results are usually represented by a binary vector (or profile) which represents the presence or absence of corresponding side effects. Potential side effect prediction methods (Cheng's method) utilize the known side effects as well as drug-related features to predict missing or undetected side effects. Liu’s method can be used for the side effect profile prediction as well as the potential side effect prediction.

Since our work is to predict the side effect profile, Pauwels’s method, Mizutani’s method and Liu’s method are adopted as the benchmark methods for the fair comparison. Here, we execute the R source codes of Pauwels’s method [http://cbio.ensmp.fr/~yyamanishi/side-effect/] and Mizutani’s method [http://web.kuicr.kyoto-u.ac.jp/supp/smizutan/target-effect/] to obtain various metric scores. We implement Liu’s method by following the details of the publication [10]. We only utilize the drug-related biological features and chemical features to predict side effect profiles, and the feature ‘known side effects’ are not used. Since benchmark methods construct the prediction models based on specific datasets and specific features, we construct our models based on the same datasets and the same features for the fair comparison. In this way, our method can be compared with benchmark methods under the same conditions. The performances of different methods are demonstrated in Table 7. In terms of AUPR, our method makes obvious improvements over Pauwels’s method, Mizutani’s method and Liu's method. Moreover, our method produces better performances in terms of multi-label learning metrics (Hamming Loss, ranking loss, one error, coverage and average precision). Reducing noise in features and combining various features are major reasons for our superior performances. In conclusion, our method can produce better performances than state-of-the-art methods.

**Table 7:** Performances of the proposed ensemble methods and state-of-the-art methods

Dataset	Method	AUPR	Hamming loss	Ranking loss	One error	Coverage	Average precision
Pauwels’s dataset	Pauwels’s method	0.3883	0.0577	0.0827	0.1779	832.7827	0.4616
	ensemble method	0.4286	0.0454	0.0737	0.1689	790.6261	0.4925
Mizutani’s dataset	Mizutani’s method	0.4107	0.0557	0.0888	0.1854	862.9757	0.4795
	ensemble method	0.4504	0.0500	0.0761	0.1657	809.6672	0.5012
Liu’s dataset	Liu's method	0.2514	0.0721	0.0927	0.9291	837.4579	0.2610
	ensemble method	0.4802	0.0524	0.0703	0.1202	795.9435	0.5134

### Independent experiment and case study

In this section, we carry out the independent experiment to evaluate the usefulness of the proposed method for practical applications. In the SIDER 4 dataset, 771 drugs which are included in Liu’s dataset are used as the training set, and 309 newly added drugs are used as the testing set. The prediction model is trained on the training drugs, and then makes predictions for the testing drugs.

In the training, prediction model selects 69 enzyme dimensions, 127 pathway dimensions, 393 target dimensions, 40 transporter dimensions, 372 indication dimensions and 315 substructure dimensions. As mentioned, the selected feature dimensions may indicate some close associations between biological (or chemical) components and drug activities. Here, we take the feature ‘transporter’ for analysis. 40 selected transporter dimensions represent 40 transporter proteins, and we manually check the selected transporter proteins in the Uniprot database and DrugBank database. We find out that 8 transporter proteins have the clear drug-related annotations and may play roles in the drug activities. For example, the transporter protein ‘P33527’ may participate directly in the active transport of drugs into subcellular organelles or influence drug distribution indirectly, and confers resistance to anticancer drugs. The supporting information about transporter proteins is provided in the Additional file 1.

When making predictions for testing drugs, the prediction model yields the AUPR score of 0.311 and AUC of 0.872. For comparison, we also implement the 5-CV cross validation on training drugs, producing the AUPR score of 0.477 and AUC of 0.875. The AUPR score in the independent experiment is less than that on the training set. The statistics on the training drugs and testing drugs shows that the average numbers of side effects for two sets of drugs are 125 and 69 respectively. The newly added drugs are recently approved drugs or experimental drugs, and only partial side effects are known. The less positive instances in the testing set lead to the lower AUPR score. For this reason, we pay attention to the recall which represents the capability of recognizing real side effects. For each testing drug, we take the top 100 scored side effect terms as the predicted side effects, and then compute the recall scores. We take drug ‘4-ap’ (DB06637) as an example to demonstrate the prediction capability of the prediction model. There are 36 known side effects for ‘4-ap’, and our model can successfully predict 32 side effects. The average of recall scores for 309 drugs is 0.463 (0.609 for top 200). As far as we know, an important goal of developing computational methods is to reduce candidates for the wet experiment. The prediction results demonstrate that we can find out 46.3 % side effects of a drug on average by biologically verifying 100 highly scored side effects terms. The numbers of correctly predicted side effects for testing drugs, the recall scores and precision scores are provided in the Additional file 1.

## Conclusions

This paper transforms the side effect prediction as a multi-label learning task. We propose a novel multi-label learning method for side effect prediction, named ‘feature selection-based multi-label k nearest neighbor’ (FS-MLKNN). FS-MLKNN can produce high-accuracy performances as well as the explainable results which help to reveal potential causes of side effects. In order to combine various features effectively, we construct individual feature-based FS-MLKNN models and use them as base predictors. Then, we combine base predictors by using the weighted scoring ensemble strategy, and develop the final prediction models for drug side effect prediction. Compared with the state-of-the-art methods, the ensemble method produces much better performances on the benchmark datasets. In conclusion, the proposed FS-MLKNN and the ensemble method are promising tools for predicting drug side effects.

### Availability of supporting data

Pauwels’s dataset is publicly available at [http://cbio.ensmp.fr/~yyamanishi/side-effect/]; Mizutani’s dataset is publicly available at [http://web.kuicr.kyoto-u.ac.jp/supp/smizutan/target-effect/]. Liu’s dataset, SIDER 4 dataset and Matlab source codes for our models are included in Additional file 1.

## Additional file

Additional file 1
**Datasets and source codes.** (ZIP 13491 kb)

## Abbreviations

MLKNN: Multi-label k-nearest neighbor method
GA: Genetic algorithm
FS-MLKNN: Feature selection-based multi-label k-nearest neighbor method
InChI key: IUPAC International Chemical Identifier
5-CV: 5-fold cross validation
AUC: Area under ROC curve
AUPR: Area under the precision-recall curve
