# Supplementary material for: Predicting drug side effects by multi-label learning and ensemble learning
Source: BMC Bioinformatics. 2015 Nov 4;16:365. doi: 10.1186/s12859-015-0774-y (PMC4634905; doi:10.1186/s12859-015-0774-y)
Supplement: Additional file 1 — Datasets and source codes. (ZIP 13491 kb) [file 12859_2015_774_MOESM1_ESM.zip › source codes and datasets 2015-09-05.zip!/source codes and datasets 2015-09-05/Guideline.doc]

**Guideline**

This *Matlab* package is for paper 'Predicting drug side effects by multi-label learning and ensemble learning'.

1. Liu’s dataset and the source codes for cross validation experiments are in the file folder ‘Liu dataset and cross validation experiment’.
2.1 The file folder “Liu dataset and cross validation experiment\Liu's dataset” contains the Liu’s dataset and the description.
2.2 The file folder “Liu dataset and cross validation experiment\source code for main experiment on Liu's dataset” contains the source codes for the main experiments on Liu’s dataset
2. SIDER 4 dataset and the source codes for independent experiment are in the file folder “SIDER4 dataset and independent experiment“.
2.1 The file folder “SIDER4 dataset and independent experiment\SIDER 4 dataset“ contains the dataset used in the paper and data description.
2.2 In file folder “SIDER4 dataset and independent experiment\source codes for independent experiment”, the file ‘independent_experiment’ is to implement the independent experiment.
2.3 The analysis on the independent experimental results are included in the file folder “SIDER4 dataset and independent experiment\independent experimental results and examples“.

***********We analyze the transport proteins selected by FS-MLKNN, and the support information for the associations with drugs is given in the “annotation on selected transporters.xls “.

-***********The prediction results for testing drugs are given in ‘prediction results for testing drugs.xls’.
